# The Sigmoidal Pregnancy: A Rare Entity Complicated by an Inappropriate Medical Intervention

**DOI:** 10.1155/2020/2725975

**Published:** 2020-04-01

**Authors:** Antoine Naem, Baraa Ahmad, Bashar Al-Kurdy

**Affiliations:** ^1^Faculty of Medicine of Damascus University, Damascus, Syria; ^2^University Hospital of Obstetrics and Gynecology of Damascus, Damascus, Syria

## Abstract

The abdominal ectopic pregnancy is very rare and accounts for less than 1% of all ectopic gestations. Due to the lack of clinical suspicion, many obstetricians confuse it with other diseases and manage it inappropriately. Here, we are reporting the case of a 41-year-old woman that was referred to our hospital because of severe vaginal bleeding caused by multiple uterine perforations during the dilatation and curettage. Her medical history was significant for constipation and a misdiagnosis of incomplete abortion. Upon the surgical exploration, a big amount of blood clots was taken out, and a fetus with his placenta inserted into the sigmoid colon were observed. The uterus and the sigmoid colon were resected. Ultimately, the patient recovered uneventfully. To the best of our knowledge, this is the third case of pure sigmoidal pregnancy and the first one to reach an advanced gestational age.

## 1. Introduction

The abdominal pregnancy is a rare form of ectopic gestations which occurs once in 10,000 live births [1] and accounts for less than 1% of ectopic pregnancies [2]. It is defined by the placental implantation into the peritoneal cavity, excluding the fallopian tubes, ovaries, and the interligamentous space. Primary abdominal pregnancy refers to the direct implantation of the fertilized ovum into the parietal or visceral peritoneum without previous attachments to the reproductive tract. Whereas the secondary abdominal pregnancy is the result of tubal abortion or the rupture of an intrauterine pregnancy into the peritoneal cavity. In general, the most common site of ectopic pregnancies is the fallopian tube as it accounts for 97% of the ectopic localizations. However, the posterior cul-de-sac seems to be the most common site of the primary abdominal pregnancy [2]. The ectopic pregnancy is a serious obstetric emergency that requires special medical attention as its maternal mortality rates can reach 20% [3]. The high mortalities could be attributed to the intensive hemorrhage provoked by separating the placental tissue from its insertion [4]. The variety of unspecific symptoms often hardens the diagnosis. The patients' complains usually range from acute abdominal pain and vaginal bleeding to mild pain and breast discomfort [5]. The diagnostic key of the abdominal pregnancy is the high clinical suspicion at the first place. In addition, the correlation between *β*-HCG levels and the radiologic imaging methods is crucial to achieve the diagnosis. Here, we present a rare case of a sigmoidal abdominal pregnancy that was misdiagnosed previously as incomplete abortion and complicated by several iatrogenic uterine perforations. To the best of our knowledge, this is the third sigmoidal pregnancy in literature and the first to reach 18 weeks of gestational age.

## 2. Case Presentation

**Figure 1:**
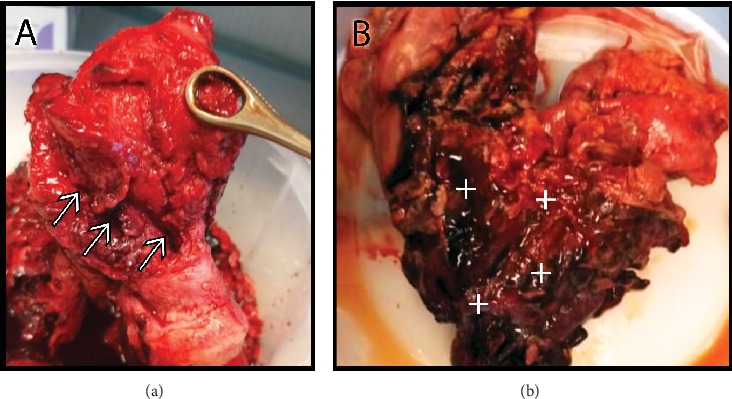
(a) The resected uterus. Note the large perforations on its right wall (white arrows). (b) The resected sigmoid colon with the placental insertion (+). Note the aggressive placental invasion of the sigmoid colon provoking severe malformation.

A 41-year-old G1P0 woman was referred to the emergency department of our hospital because of severe vaginal bleeding. As the patient was unconscious, her medical history was taken from her accompanying sister. The patient's history was significant for 18 weeks of amenorrhea and positive pregnancy tests, in addition to constipation and a misdiagnosis of incomplete abortion. As a result of the wrong diagnosis, an external obstetrician performed a dilatation and curettage (D&C) that ended up in perforating the uterus. Upon the clinical examination, the patient looked pale, her pulse was weak, her heart rate was 140 beats per minute, and her blood pressure was 80/40 mmHg. The transabdominal ultrasonography showed an empty heteromorphic uterus and excessive amount of free intra-abdominal fluid. Therefore the diagnosis of a perforated uterus was confirmed, and a ruptured ectopic pregnancy was suspected. The patient was moved immediately to the operating room to perform an emergent exploratory laparotomy and resuscitated with 6 full-blood units and 5 plasma units. A Pfannenstiel incision was made; the abdominal muscles and fascia were dissected. Upon reaching the peritoneal cavity, a big amount of blood clots was taken out and a fetus with his placenta inserted exclusively into the sigmoid colon were observed. The uterus was perforated in different locations on the contralateral side of the placental insertion. In addition, ileal and appendicular injuries were also observed. So the diagnosis of an abdominal pregnancy was achieved intraoperatively. The fetus was taken out, and the internal iliac arteries were ligated to reduce the hemorrhage. A hysterectomy was done due to the multiple large defects of the uterine wall. Regarding the wide placental insertion on the sigmoid colon and the potential risk of inducing additional hemorrhage by dissecting it, a sigmoidectomy was considered as the ultimate management. Therefore, the incision was dilated longitudinally superior to the umbilicus, and the sigmoid colon was resected (Figure 1). The descending colon was isolated, and a skin colostomy was made.

**Figure 2:**
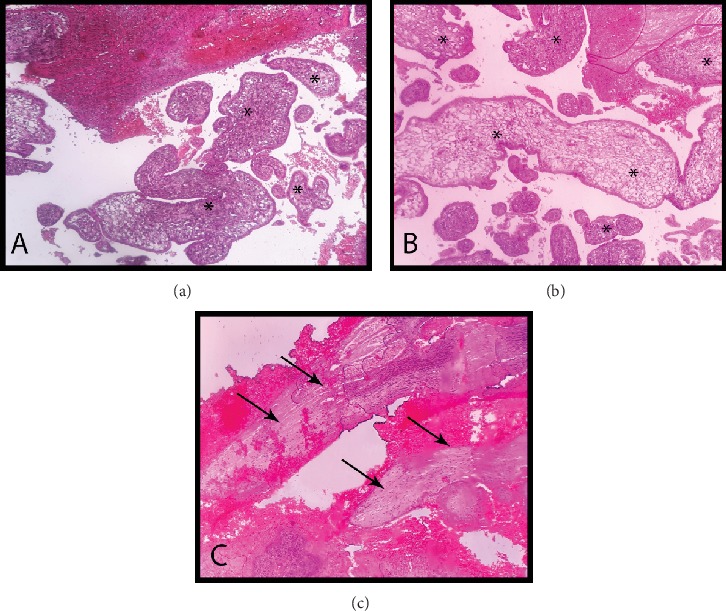
The pathologic appearance of the resected specimen stained in hematoxylin and eosin. (a, b) Chorionic villi (^∗^) invading the sigmoid wall. (c) The intensive hemorrhage within the sigmoid wall between its smooth muscle layers (arrows) that was provoked by the ectopic placental insertion.

Finally, the intestinal injury was repaired before skin closure. The operation lasted for 7 hours. The patient's vital signs returned to their normal limits after the surgery and she was stable during the eight days of follow-up. The pathologic examination of the resected specimen showed the presence of normal chorionic villi invading the sigmoid wall (Figure 2). However, due to the D&C, the endometrium was not fully evident when the uterus was examined microscopically. The endometrial remnants showed Arias-Stella reaction in the endometrial glands.

## 3. Discussion

The abdominal pregnancy occurs when the conceptus implants within the peritoneal cavity far from the ovaries, fallopian tubes, and the interligamentous space. The fertilized ovum seems to be able to implant exclusively into the parietal or visceral peritoneum and occasionally into islands of endometriosis [6]. The abdominal ectopic pregnancy is subdivided into primary and secondary. Primary abdominal pregnancy happens when the blastocyst implants directly into the peritoneal cavity without previous attachments to the uterus or fallopian tubes. While secondary abdominal pregnancy is the result of tubal abortion or the rupture of an intrauterine pregnancy with subsequent implantation in the abdomen. In 1942, Studdiford created three criteria to differentiate between these two types of pregnancy, which are “1) The two ovaries and tubes are normal without any evidence of recent or remote tubal injury. 2) The absence of any evidence of a uteroperitoneal fistula. 3) The presence of a pregnancy related exclusively to the peritoneal surface and early enough to eliminate the possibility of primary tubal nidation” [7]. Obviously, diagnosing the primary abdominal pregnancy using the mentioned criteria is based on eliminating any chance of a previous tubal or ovarian pregnancy. However, these criteria still have its limitations in categorizing the abdominal pregnancies. As noticed, the first two criteria tend to determine the uterine and tubal validity through gross inspection. The macroscopic examination carries a low diagnostic value because it might fail to detect a precedent primary tubal nidation. In addition, the microscopic evidence of a primary tubal pregnancy even when the tube appeared normal grossly was reported in the literature [6]. Therefore, the most confident way to investigate the tubal and uterine validity is the pathologic examination. However, this is not fully accepted recently, because the general intentions are now tending to preserve the maternal fertility when possible; making the uterus and tubes unavailable for the pathologic examination.

Another limitation of the Studdiford criteria is the unreliability of the gestational age to differentiate between primary and secondary abdominal pregnancies. When the ovum starts its mitotic activity, its nutritional requirements increase. Therefore, losing the placental blood supply by the separation of the developing placenta may harm the embryo and lead to its death, which is inconvenient with achieving a secondary abdominal implantation [8]. On this basis, we suggest that the secondary implantation after complete tubal abortion should happen also at an early gestational age and that contradicts the third criterion of Studdiford. Some authors considered the retention of the placental blood supply in the incomplete abortion an important survival factor for achieving a secondary implantation [6]. By taking the previous reasons in consideration, excluding the tubal abortion upon investigating its three forms that were described by Caspi and Sherman is highly recommended for categorizing the abdominal pregnancies [8]. In our case, we could not confirm the pregnancy type as we were unable to isolate the fallopian tubes due to the intense adhesions that were found within the abdomen.

The most common locations of the abdominal pregnancy are the posterior cul-de-sac, the mesosalpinx, and the omentum [2]. In fact, there are several reasons that make the sigmoid colon an elected implantation site, such as its intraperitoneal localization and the presence of the sigmoid mesocolon that may be prolonged in some patients making it closer to the ovaries. However, the small number of sigmoidal pregnancies could be explained by the relatively low vasculature of the sigmoid colon that may not meet the conceptus nutritional needs. Interestingly, altered defecating habits like increased defecating frequency [9] or constipation [10, 11] were noted to be exclusive to the abdominal pregnancy where chorionic villi invade the sigmoidal wall. This could be explained either by the inflammatory irritation caused by the implantation that increases the bowl motility or by damaging the myenteric plexus (Auerbachian plexus) causing localized ileus and a subsequent bowl obstruction. Therefore, these symptoms may refer to a sigmoidal pregnancy in patients with ectopic pregnancy of unknown location.

However, the high correlation between radiologic imaging methods and the laboratory pregnancy test is crucial to diagnose the abdominal pregnancy. In general, transvaginal ultrasonography and transabdominal ultrasonography are the most favorable initial imaging techniques [12]. When the *β*-HCG serum levels are elevated, with the presence of an empty uterus and adnexa, a pan-abdominal ultrasonographic scan is advisable. Occasionally, the computed tomography scan could be also used. In emergent cases, the presence of an empty uterus with free intra-abdominal fluid, accompanied by amenorrhea or elevated *β*-HCG serum levels above the discriminating zone, is a great indication of performing an exploratory laparotomy. Whether it is laparoscopy or laparotomy depends on the surgeon's experience and patient's stability. It is noteworthy that the negativity of the *β*-HCG test cannot exclude the diagnosis because more than 1% of the ectopic pregnancies may not have elevated *β*-HCG levels [13].

As the massive bleeding from the implantation site remains the major cause of maternal death [4], many authors have applied impressive hemostatic techniques to control the hemorrhage. The electric cauterization is a simple method to control the hemorrhage, but thermal injury could be provoked to the adjacent organs when the placental insertion is vast. Therefore, at early pregnancy, the vasopressin injection within and around the gestational sac is a good technique to minimize the hemorrhage after resecting the conceptus [14]. Whereas at advanced stages, applying the gelatin-thrombin matrix [15] is more suitable because it avoids the side effects that might be caused by the high dosage of the injected vasopressin. However, the early detection of the sigmoidal pregnancy minimizes the risks of the surgical procedure, and the gestational sac could be removed more easily using the traditional surgical methods [9, 11].

## 4. Conclusion

The primary abdominal pregnancy is a rare clinical finding that may be encountered once in a lifetime, but when it's encountered, it should be dealt with firmly. Although the majority of patients present with unspecific symptoms, the altered defecating habits like constipation may hide a sigmoidal pregnancy behind. The good correlation between the ultrasonographic findings and the *β*-HCG levels is crucial to achieve the diagnosis. However, the entire abdomen should be scaned by either ultrasonography or computed tomography scan. The management should be minimally invasive and well planned in order to prevent the massive hemorrhage that may cost the patient her life. In emergencies, performing an exploratory laparotomy or laparoscopy combined with less traumatic hemostatic technique is favorable. Finally, when the D&C is needed, it should be delayed until the patient is stable again.

## Abbreviations

D&C: Dilatation and curettage
*β*-HCG: Beta subunit of the human chorionic gonadotropin.
